# Associations between abrupt transition, dialysis-requiring AKI, and early mortality in ESKD among U.S. veterans

**DOI:** 10.1186/s12882-023-03387-9

**Published:** 2023-11-14

**Authors:** Raymond K. Hsu, Anna D. Rubinsky, Michael G. Shlipak, Kirsten L. Johansen, Michelle M. Estrella, Benjamin J. Lee, Carmen A. Peralta, Chi-yuan Hsu

**Affiliations:** 1 Division of Nephrology, Department of Medicine, University of California, San Francisco, San Francisco, CA USA; 2 Department of Epidemiology and Biostatistics, University of California, San Francisco, San Francisco, CA USA; 3 Department of Medicine, Kidney Health Research Collaborative, San Francisco Veterans Affairs Medical Center, San Francisco, CA USA; 4 Chronic Disease Research Group, Hennepin Healthcare Research Institute, Minneapolis, MN USA; 5 Division of Nephrology, Hennepin Healthcare, Minneapolis, MN USA; 6 Houston Methodist Institute for Academic Medicine, Houston, TX USA; 7 Houston Kidney Consultants, Houston, TX USA; 8 Cricket Health, Inc, San Francisco, CA USA

**Keywords:** Abrupt transition, AKI, Early mortality in ESKD

## Abstract

**Background:**

Mortality is high within the first few months of starting chronic dialysis. Pre-ESKD trajectory of kidney function has been shown to be predictive of early death after dialysis initiation. We aim to better understand how two key aspects of pre-dialysis kidney function—an abrupt transition pattern and an episode of dialysis-requiring AKI (AKI-D) leading directly to ESKD—are associated with early mortality after dialysis initiation.

**Methods:**

We extracted national data from U.S. Veterans Health Administration cross-linked with the United States Renal Data System (USRDS) to identify patients who initiated hemodialysis during 2009–2013. We defined abrupt transition as having a mean outpatient eGFR ≥ 30 ml/min/1.73m^2^ within 1 year prior to ESKD. AKI-D was identified using inpatient serum creatinine measurements (serum Cr increase by at least 50% from baseline) along with billing codes for inpatient receipt of dialysis for AKI within 30 days prior to the ESKD start date. We used multivariable proportional hazards models to examine the association between patterns of kidney function prior to ESKD and all-cause mortality within 90 days after ESKD.

**Results:**

Twenty-two thousand eight hundred fifteen patients were identified in the final analytic cohort of Veterans who initiated hemodialysis and entered the USRDS. We defined five patterns of kidney function decline. Most (68%) patients (*N* = 15,484) did not have abrupt transition *and* did not suffer an episode of AKI-D prior to ESKD (reference group). The remaining groups had abrupt transition, AKI-D, or both. Patients who had an abrupt transition with (*N* = 503) or without (*N* = 3611) AKI-D had the highest risk of early mortality after ESKD onset after adjustment for demographics and comorbidities (adjusted HR 2.10, 95% CI 1.66–2.65 for abrupt transition with AKI-D; adjusted HR 2.10, 95% CI 1.90–2.33 for abrupt transition without AKI-D). In contrast, patients who experienced AKI-D without an abrupt transition pattern (*N* =  2141 had only a modestly higher risk of early death (adjusted HR 1.19, 95% CI 1.01–1.40).

**Conclusions:**

An abrupt decline in kidney function within 1 year prior to ESKD occurred in nearly 1 in 5 incident hemodialysis patients (18%) in this national cohort of Veterans and was strongly associated with higher early mortality after ESKD onset.

## Background

High death rates within the first few months after starting chronic dialysis is a significant problem in the U.S. and worldwide [1–4]. According to data from the United States Renal Data System (USRDS), in the first two months after starting chronic hemodialysis, all-cause mortality reaches as high as 200/1000 and 600/1000 patient-years among patients aged below and above 65 years respectively [5].

Older studies investigating risk factors for mortality early after maintenance dialysis initiation did not have available information about pre-dialysis clinical course [1–4]. In the past decade however, it has become clear that patients’ pre-dialysis kidney function trajectory as they transition to ESKD is important to their early prognosis with ESKD. Specifically, higher mortality rates have been observed among those patients who transition to ESKD after hospitalized dialysis-requiring acute kidney injury (AKI-D) [6, 7], or after abrupt transition caused by rapidly declining kidney function preceding the start of dialysis [8–12].

These two subgroups of patients presumably overlap partially, since some patients who transition to ESKD abruptly presumably did so because of AKI-D [9]. But previous studies have not evaluated for differences in prognosis among those who transition abruptly with or without AKI-D; nor have studies defined the relative sizes of these subpopulations. To fill these gaps in the literature, we conducted the current analysis using U.S. national data from the Veterans Health Administration (VHA).

## Methods

### Study population

**Fig. 1:**
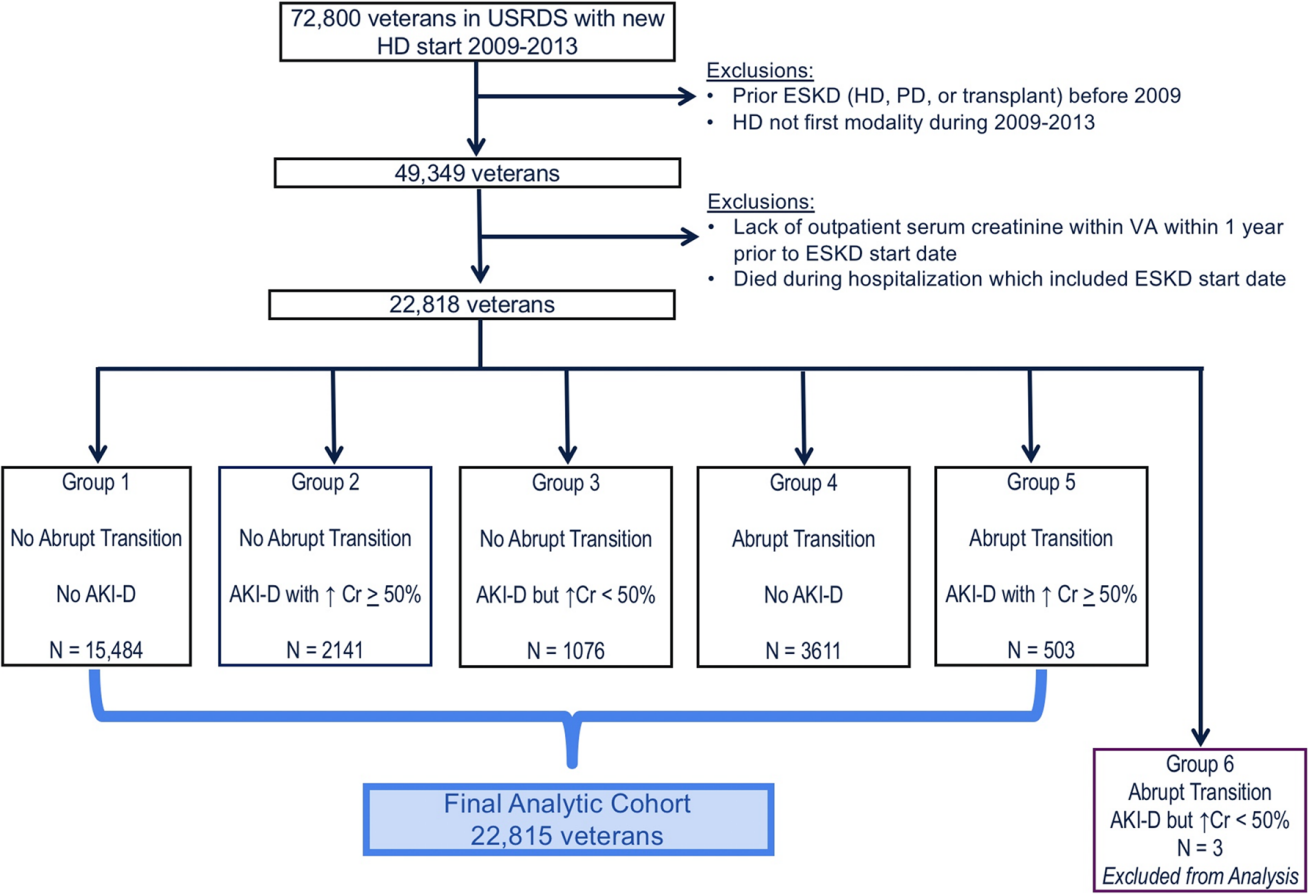
Derivation of study population and delineation based on patterns of transition. USRDS: United States Renal Data System. ESKD: end-stage kidney disease. HD: hemodialysis. PD: peritoneal dialysis. AKI-D: dialysis-requiring acute kidney injury. Cr: creatinine

We used data from the VHA and cross-linked with the USRDS to identify all adult Veterans (age 18 or older) who initiated hemodialysis for ESKD from October 1, 2009 to September 30, 2013. Only incident hemodialysis patients were included. We chose to exclude ESKD patients who initiated peritoneal dialysis or received a kidney transplant, as rates and determinants of death may differ by modality. We excluded patients for whom there were no outpatient serum creatinine measurements within 7–365 days prior to the ESKD start date, as we needed to establish the pre-dialysis mean outpatient eGFR within that time frame. This approach also ensured that the final analytic cohort consisted of patients who were obtaining some clinical care at the VA. Figure 1 illustrates the derivation of our study population.

Written informed consent was waived by the University of California, San Francisco (UCSF) Institutional Review Board, who waived the informed consent due to the retrospective nature of the study (IRB No. 15–17546). All methods were carried out in accordance with IRB guidelines.

### Definition of abrupt transition based on observed pre-dialysis mean eGFR values

Pre-dialysis mean outpatient eGFR values (from within 7–365 days prior to ESKD) were derived from the observed serum creatinine measurements taken at a VA facility, excluding measurements during acute care hospitalizations or emergency department visits [13]. *“Abrupt transition”* is defined as having pre-dialysis mean eGFR of ≥ 30 ml/min/1.73m^2^ calculated from observed data within the 7–365 day period. We adopted this definition based on prior studies and data suggesting that this cutoff as a predictor of post-dialysis mortality [14, 15].

### Definition of hospitalized dialysis-requiring AKI (AKI-D) leading to ESKD

Hospitalized cases of AKI-D are those that met all of the following criteria: VA acute care hospitalization that lasted at least 24 h, receipt of dialysis during admission (captured using ICD-9 procedure code 39.95 or Current Procedural Terminology (CPT) codes 90935, 90937, 90945, 90947 and 90999 [16–18]), and a rise in serum creatinine during the hospitalization by 0.3 mg/dL or by at least 50% relative to the pre-hospitalization baseline creatinine [19] calculated as the mean outpatient creatinine between 7 to 365 days before each hospitalization [20]. In order for AKI-D to qualify as directly leading to ESKD, the ESKD start date listed in USRDS must fall within the AKI-D hospitalization dates or within 30 days after AKI-D hospital discharge.

This definition of AKI based on serum creatinine is the same as that used in the National Institute of Health sponsored ASsessment, Serial Evaluation, and Subsequent Sequelae of Acute Kidney Injury (ASSESS-AKI) study [21]. However, it is possible that those who only met the 0.3 mg/dL definition are actually patients with progressive CKD who had an inpatient dialysis start for ESKD. We thus separated out the AKI-D individuals with a ≥ 50% rise in creatinine from those without.

### Primary predictor: patterns of transition to ESKD

Based on the components described above, the study population could be divided into the following 6 subgroups (Fig. 1) based on the pattern of transition to ESKD:

1. No abrupt transition (mean outpatient eGFR < 30 ml/min/1.73m2) and no AKI-D (reference group): *N* = 15,484
2. No abrupt transition (mean outpatient eGFR < 30 ml/min/1.73m2) but experienced AKI-D with ≥ 50% rise in serum creatinine during AKI-D episode: *N* = 2141
3. No abrupt transition (mean outpatient eGFR < 30 ml/min/1.73m2) but met definition of AKI-D with > 0.3 mg/dL (but < 50%) rise in serum creatinine during AKI-D episode: *N* = 1076
4. Abrupt transition (mean outpatient eGFR ≥ 30 ml/min/1.73m2) and no AKI-D: *N* = 3611
5. Abrupt transition (mean outpatient eGFR ≥ 30 ml/min/1.73m2) and experienced AKI-D with ≥ 50% rise in serum creatinine during AKI-D episode: *N* = 503.
6. Abrupt transition (mean outpatient eGFR ≥ 30 ml/min/1.73m2) and met definition of AKI-D with > 0.3 mg/dL (but < 50%) rise in serum creatinine during AKI-D episode: *N* = 3.

Owing to the small size of the last group, those three patients were not included in the final analytic cohort (Fig. 1). The final analytic cohort thus consisted of 22,815 veterans from the 5 remaining subgroups.

### Primary outcome: all-cause 90-day mortality

Our primary outcome was all-cause mortality within 90 days of ESKD start date, ascertained using the VA Vital Status Files and data from the USRDS.

### Covariates

Demographic characteristics included age, sex, and race/ethnicity. Comorbidities were extracted using ICD-9-CM diagnostic codes up to 4 years prior to the study period and included diabetes, hypertension, coronary disease, peripheral arterial disease, cerebrovascular disease, chronic lung disease, moderate to severe liver disease. Most recent body mass index (BMI) calculated from weight and height, and mean outpatient systolic blood pressure (SBP) within one year prior to dialysis initiation were also extracted from VA data sources.

We additionally ascertained the following care practice variables for exploratory analyses. VA care was defined as the number of outpatient visits to any VA facility within the past year prior to ESKD. CKD care was defined as the number of nephrology visits within the four years prior to ESKD. Vascular access was defined according to the first access used for maintenance hemodialysis as listed in the USRDS Medical Evidence files.

### Statistical analysis

We used Cox models to determine the association between the pre-ESKD kidney function trajectory patterns of transition to ESKD and mortality within 90 days. We assessed the proportional hazards assumption by testing the Schoenfeld residuals, examining plots of the Schoenfeld residuals over time, examining log–log plots (graph of the log(-log(survival)) versus log of survival time, comparing Kaplan–Meier observed survival curves with predicted curves from the Cox model, and testing for time-dependent covariates. Plots of the Schoenfeld residuals showed no trend over time, and tests of Schoenfeld residuals were not statistically significant. In addition, examination of log–log plots and plots of Kaplan–Meier observed vs predicted survival revealed no evidence of violation of the proportional-hazards assumption, and tests for time-varying covariates were not statistically significant.

We designated the reference group as those who transitioned to ESKD without abrupt transition (i.e., with a pre-dialysis baseline eGFR < 30 ml/min/1.73m^2^) and without a preceding AKI-D event. The final multivariable adjusted model incorporated demographics, comorbidities, BMI, and SBP as covariates.

We also performed exploratory analyses adding the following care practice predictors to the final adjusted model: number of nephrology visits within 4 years, number of any VA outpatient visits within 1 year, and first vascular access type to determine the degree to which these care practice factors may attenuate associations of transition patterns with increased risk of early death. These measures of clinical care could be mediators for any observed association between abrupt transition or AKI-D and higher rates of mortality. Analyses were performed using STATA (TX: Statacorp, LLC).

## Results

Among the final analytic cohort of 22,815 patients, the median age was 69 years old (interquartile range 62–79); 98% were male, 27% were Black, and 6% were of Hispanic ethnicity. Table 1 shows demographic and clinical characteristics by pre-ESKD kidney function trajectory patterns of transition to ESKD.

**Table 1:**
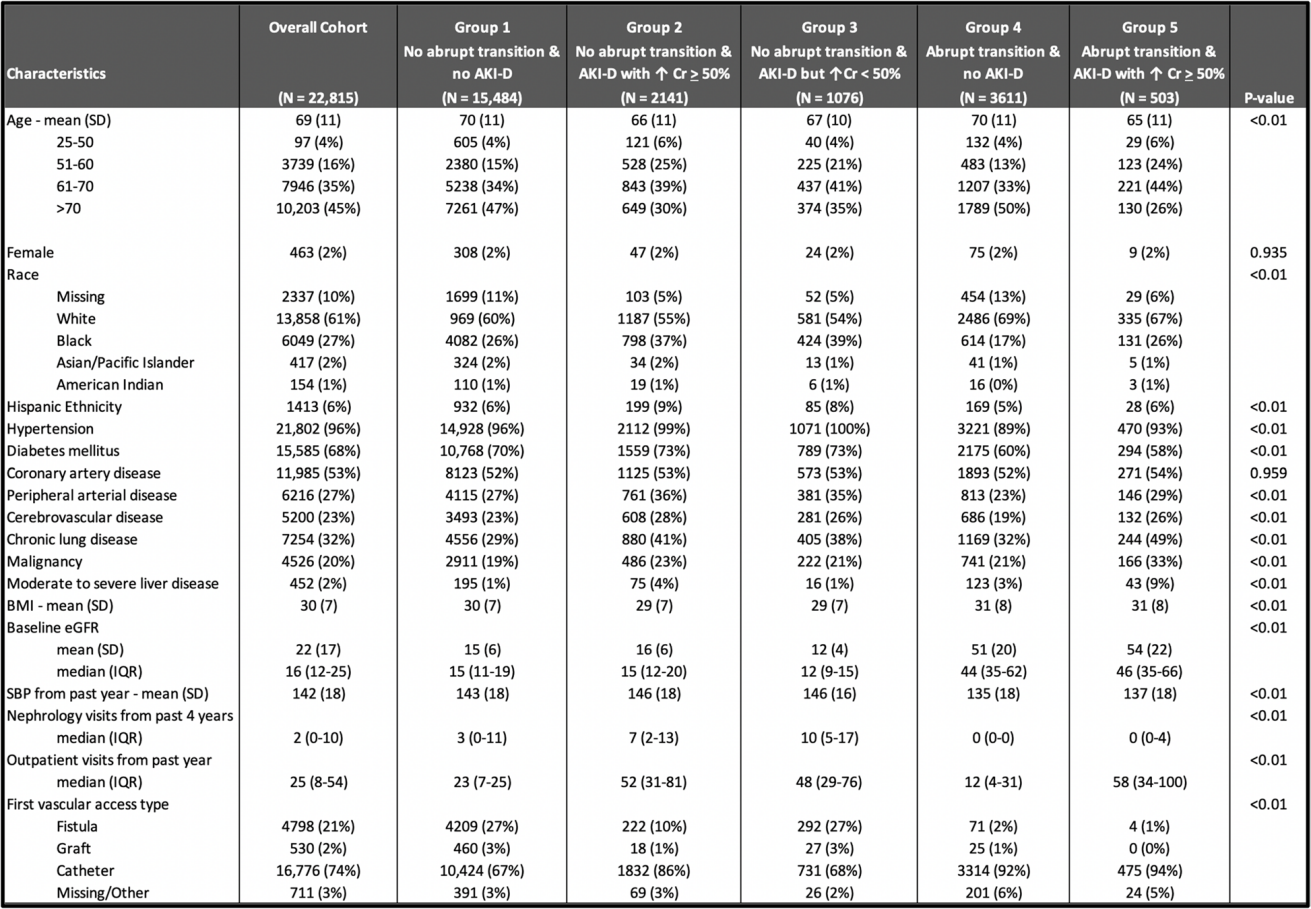
Baseline characteristics of incident hemodialysis patients by patterns of transition to ESKD

Values are numbers (percentages) unless stated otherwise

*Abbreviations*: *AKI-D* dialysis-requiring AKI, *Cr* creatinine, *SD* standard deviation, *eGFR* estimated glomerular filtration rate, *IQR* interquartile range

The largest subgroup was those who transitioned to ESRD without meeting the criteria for abrupt transition or any definitions of AKI-D (*N* = 15,484 or 68% of the analytic cohort)(Group 1). There were 4,114 patients (18%) who met criteria for abrupt transition; and 2644 patients (12%) who met the definition of AKI-D with ≥ 50% rise in serum creatinine. Only 503 patients (%) had both abrupt transition and AKI-D.

There were 2175 deaths during the first 90 days after initiation of chronic hemodialysis, corresponding with crude all-cause mortality rate of 395 per 1000 patient-years. In unadjusted models, abrupt patterns of transition with or without AKI-D were associated with higher risk of all-cause mortality within 90 days of ESKD start, compared with the reference group with no abrupt transition and no AKI-D (hazard ratio [HR] 2.41, 95% CI 2.19–2.65 for abrupt transition without AKI-D and 2.14, 95% CI 1.71–2.68 for abrupt transition with AKI-D) (Table 2).

**Table 2:**
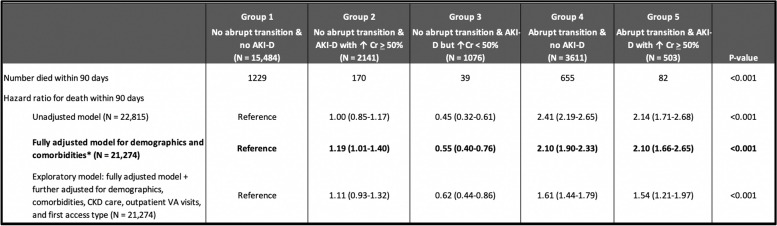
90-day mortality in incident hemodialysis patients by patterns of transition to ESKD

*Model adjusted for age, sex, race, ethnicity, diabetes, coronary artery disease, peripheral artery disease, cerebrovascular disease, chronic lung disease, malignancy, moderate to severe liver disease, systolic blood pressure, and body mass index

In final models which controlled for demographics and comorbid conditions (Table 2), similar results were seen: the presence or absence of AKI-D did not appear to affect the strengths of associations between abrupt transition and all-cause deaths within 90 days of ESKD start (adjusted HR was 2.10 in both the presence and in absence of AKI-D; both *p* < 0.001). AKI-D itself without abrupt transition had weaker (albeit still statistically significant) association (adjusted HR 1.19, 95% CI 1.01–1.40).

The strengths of association between abrupt transition and all-cause deaths were attenuated but remained statistically significant in exploratory analysis (Table 2) which further adjusted for number of nephrology visits, number of any VA outpatient visits, and first vascular access type (adjusted HR was 1.61 without and 1.54 with AKI-D; both *p* < 0.001). In these models, the association of AKI-D only with mortality was mildly attenuated.

Of note, individuals classified as meeting the criteria for AKI-D solely on the basis of a ≥ 0.3 mg/dL (but not ≥ 50%) rise in serum creatinine and without abrupt decline of kidney function (Group 3) actually had a lower risk of all-cause mortality within 90 days of ESKD start (unadjusted HR 0.45, 95% CI 0.32–0.61). This association persisted in fully adjusted & exploratory models (Table 2).

## Discussion

In this national study analyzing data from the Veterans Health Administration (VHA), we characterized distinct patterns of kidney function decline leading to incident ESKD and examined the associations between patterns of transition to ESKD with early mortality after dialysis initiation. We evaluated the joint contributions to mortality risk of both the abruptness of kidney function decline, defined as a mean outpatient eGFR ≥ 30 ml/min/1.73m^2^ in the year prior to ESKD, and the presence of AKI-D leading directly to ESKD..

We showed that 6255 out of 22815 (27%) in the analytic cohort met criteria for having abrupt kidney function decline and/or having experienced AKI-D including ≥ 50% rise in serum creatinine and a new start of dialysis during a hospital admission immediately preceding ESKD. This is consistent with published literature showing that non-linear declines in kidney function are common prior to ESKD [6–12, 22], in contrast to the earlier paradigm that kidney disease progresses at a uniform rate [23]. These non-linear progression patterns suggest that it is often challenging to predict which patients will develop ESKD over a given time frame. One possible implication is that policy makers should consider the heterogenous natural histories of kidney disease progression when setting quality metrics such as the proportion of incident ESKD patients starting dialysis with a permanent vascular access or having been pre-emptively referred for kidney transplant.

Comparing our reported distribution of patterns of transition to ESKD with prior publications is not straightforward in part due to differences in case definition. For example, a prior NIH-funded prospective multicenter cohort study showed that 9% of incident hemodialysis cases had abrupt decline of kidney function, with abrupt decline defined as having a mixed-effects model estimated patient-specific eGFR at 3 months prior to initiation of hemodialysis therapy of ≥ 30mL/min/1.73m^2^ [10]. A previously published VA analysis reported that 21% of patients experienced an abrupt decline in kidney function, defined as recorded eGFR at the time of transition being > 50% lower than the eGFR value that was expected based on a mixed-effect model generated eGFR trajectory of each individual during the last year before ESKD [12]. However, those extrapolations assuming linear declines in eGFR may not be optimal when studying patients with non-linear, abrupt changes in kidney function; for that reason we only considered observed eGFR readings. A prior report based on members of a large integrated healthcare delivery system in Northern California concluded that 33% of ESKD patients started dialysis preceded by AKI-D, but AKI-D was defined only as receipt of new kidney replacement therapy during hospitalization without any requirement for acute changes in serum creatinine [6]. Finally, in a nationwide study of Medicare patients, 17% of new patients in dialysis facilities were classified as having had AKI-D, but this classification was based on administrative data [7, 24]. An advantage of the current study is that our definition of AKI-D incorporated actual changes in measured serum creatinine incorporating pre-hospitalization baseline and changes during hospitalization.

However, regardless of the exact definitions for abrupt transition or AKI-D, the consistent observation across this and prior studies has been that patterns of rapid pre-ESKD kidney function trajectory are associated with higher mortality rates after onset of dialysis, compared with reference groups with patterns of slow gradual declines in kidney function and mostly outpatient initiation of chronic dialysis. We chose to focus on mortality within the first three months of dialysis initiation in this paper in part because Healthy People 2020 [25] has as an objective to “reduce the number of deaths in dialysis patients within the first 3 months of initiation of renal replacement therapy.”

None of the prior publications, however, examined what proportion of patients classified with abrupt transition experienced AKI-D. This is in part because subdividing may not have been possible in those studies due to smaller sample size or lack of granular data such as inpatient serum creatinine values and information regarding acute dialysis procedures during hospitalization. We note that, perhaps a bit surprisingly, AKI-D as an immediate proximal precipitant to ESKD only accounted for a minority of those classified as having abrupt transition (only 503 out of 4114 or 12% when AKI was defined as ≥ 50% rise in serum creatinine).

Importantly, while both abrupt transition and AKI-D were associated with higher risk of all-cause mortality within 90 days of ESKD start date when controlling for demographics and comorbidities, the associations were stronger for abrupt transition than for AKI-D. The presence or absence of AKI-D did not appear to affect the strength of associations between abrupt transition and all-cause mortality within 90 days of ESKD start (adjusted HR was 2.10 in the full model regardless (Table 2)). Conversely, the presence or absence of abrupt transition did affect the strength of associations between AKI-D and all-cause mortality within 90 days of ESKD start (adjusted HR 2.10 with abrupt transition and 1.19 without (Table 2)). Some have hypothesized that the observation that incident ESKD patients who experienced AKI-D have worse outcomes is largely because an acute illness resulted in the AKI hospitalization and also caused the higher mortality. Our data suggest this is perhaps a less important factor than an alternative explanation: AKI-D patients do worse because they are unprepared for dialysis and start dialysis with a catheter, which is also the case for patients who transition abruptly even without AKI-D. In support of this hypothesis, patients meeting the criteria for abrupt transition had fewer pre-dialysis nephrologist visits and were more likely to use a dialysis catheter for vascular access (Table 1); adjusting for these differences attenuated the hazard ratios.

However, adjusting for vascular access and pre-dialysis care did not fully attenuate the associations, suggesting there are other pathways. Another commonality among abrupt transition patients regardless of presence or absence of AKI-D is more rapid loss of residual native kidney function after ESKD onset compared with patients who gradually progress to ESKD, who will presumably lose residual function more slowly. Residual kidney function is associated with mortality in chronic hemodialysis patients [26–28]. Nevertheless, the importance of more rigorous and multi-disciplinary care, such as “low GFR clinics” or “transition units” for advanced CKD care both before critical illness and after hospital discharge deserves emphasis, in light of the increased needs associated with accelerated kidney function decline.

The relatively lower risk of death among patients who did not meet the criteria for abrupt transition but did meet the definitions of AKI-D based on a ≥ 0.3 mg/dL (but not ≥ 50%) rise in serum creatinine was unexpected (Group 3). The exact reasons for this are unclear, but we suspect this group represented patients with advanced CKD, as they had mean pre-dialysis eGFR of 16 mL/min/1.73m^2^ during the year prior to ESKD (Table 1)). Therefore, small changes in kidney function resulted in sufficient increases in serum creatinine to meet the definition for “AKI,” but were phenotypically more representative of patients with progressive CKD who experienced inpatient dialysis initiation for ESKD, rather than “AKI-D.”

The strengths of this study include the large sample size, the nationally representative capture of patients receiving care in a single system, and the availability of comprehensive, longitudinal granular laboratory and clinical data. Because of the frequency of laboratory measurements, we did not need to assume linear trajectory of kidney function, which some prior papers had done [10]. We were able to rigorously define AKI-D using creatinine values and did not rely on diagnostic codes, which are known to have suboptimal performance characteristics.

Limitations of the study include the study population, which was only U.S. Veterans, and therefore the results may not be generalizable to other patient populations. The percentage of women was low (2%), although the absolute number of women (*N* = 463) is still larger than the absolute number of women in other published studies [10]. We don’t have a full or definitive explanation for why abrupt transition (or AKI-D) is a risk factor for worse outcome after dialysis initiation. It is possible that factors not fully captured or characterized here–such as malignancies and treatment for malignancies–explain both the AKI-D/abrupt transition and the higher rates of death [8]. We did not have granular data on dialysis prescriptions during AKI-D episodes, such as rate of ultrafiltration and modality (continuous vs. prolonged intermittent vs. traditional intermittent) which may play a role in short and long-term kidney recovery. The impact of acute dialysis management on kidney and mortality outcomes deserves further exploration in future studies. We also did not have sufficient data or resources to determine the etiologies of AKI-D or abrupt transition to include in the analyses. This is also challenging as there is no accepted gold-standard methodology to adjudicate etiology of AKI—including how to reliably distinguish between pre-renal azotemia and acute tubular necrosis [29]. Prior studies have shown poor agreement among adjudicators [30]. Some patients with underlying CKD who started dialysis and died during hospital stays were likely excluded due to lack of entry into USRDS. The exclusion of this population likely resulted in underestimation of the association between acute kidney function deterioration and mortality, and the existence of these patients highlights the importance of considering conservative, non-dialysis care for those with significant frailty and other comorbidities such as malignancy.

## Conclusions

In conclusion**,** nearly 1 in 5 incident hemodialysis patients in this national VA population had an abrupt pattern of transition to ESKD, and this was strongly associated with higher early mortality. AKI-D patients are only a minority of this subgroup, and surrogates of dialysis preparedness only partially accounted for increased early mortality. More research is needed to uncover approaches to improve outcomes (including mortality, preservation of residual kidney function, kidney recovery, and patient-reported outcomes) in this vulnerable population.

## Data Availability

Data analyzed in this study are available from the U.S. Veterans Health Administration, but restrictions apply to the availability of these data, which were used under the license for the current study and are therefore not publicly available. Data are however available from the corresponding author upon reasonable request and with permission from the Veterans Health Administration.
